# Thermoplastic Starch Biocomposite Films Reinforced with Nanocellulose from *Agave tequilana* Weber var. Azul Bagasse

**DOI:** 10.3390/polym15183793

**Published:** 2023-09-17

**Authors:** María Guadalupe Lomelí-Ramírez, Benjamín Reyes-Alfaro, Silvia Lizeth Martínez-Salcedo, María Magdalena González-Pérez, Manuel Alberto Gallardo-Sánchez, Gabriel Landázuri-Gómez, J. Jesús Vargas-Radillo, Tania Diaz-Vidal, José Guillermo Torres-Rendón, Emma Rebeca Macias-Balleza, Salvador García-Enriquez

**Affiliations:** 1 Department of Wood, Cellulose and Paper, University Center for Exact Sciences and Engineering, University of Guadalajara, km 15.5 at the Guadalajara-Nogales Highway, Zapopan 45220, Mexico; maria.lramirez@academicos.udg.mx (M.G.L.-R.); silviaiq28@gmail.com (S.L.M.-S.); magda.gonzaleziq@gmail.com (M.M.G.-P.); j.vargas@academicos.udg.mx (J.J.V.-R.); jose.torres@academicos.udg.mx (J.G.T.-R.); 2 Department of Chemical Engineering, Michoacana University of Saint Nicholas of Hidalgo, Morelia 58030, Mexico; breyesalfaro@gmail.com; 3 Department of Civil Engineering and Topography, University Center for Exact Sciences and Engineering, University of Guadalajara, Marcelino Garcia Barragan Street, Number 1451, Guadalajara 44430, Mexico; manuel.gallardo@academicos.udg.mx; 4 Department of Chemical Engineering, University Center for Exact Sciences and Engineering, University of Guadalajara, Marcelino Garcia Barragan Street, Number 1451, Guadalajara 44430, Mexico; gabriel.landazuri@academicos.udg.mx (G.L.-G.); taniadzv@gmail.com (T.D.-V.)

**Keywords:** *Agave tequilana* bagasse, cellulose nanocrystals, cellulose nanofibers, starch films

## Abstract

In this work, cellulose nanocrystals (CNCs), bleached cellulose nanofibers (bCNFs), and unbleached cellulose nanofibers (ubCNFs) isolated by acid hydrolysis from *Agave tequilana* Weber var. Azul bagasse, an agro-waste from the tequila industry, were used as reinforcements in a thermoplastic starch matrix to obtain environmentally friendly materials that can substitute contaminant polymers. A robust characterization of starting materials and biocomposites was carried out. Biocomposite mechanical, thermal, and antibacterial properties were evaluated, as well as color, crystallinity, morphology, rugosity, lateral texture, electrical conductivity, chemical identity, solubility, and water vapor permeability. Pulp fibers and nanocelluloses were analyzed via SEM, TEM, and AFM. The water vapor permeability (WVP) decreased by up to 20.69% with the presence of CNCs. The solubility decreases with the presence of CNFs and CNCs. The addition of CNCs and CNFs increased the tensile strength and Young’s modulus and decreased the elongation at break. Biocomposites prepared with ubCNF showed the best tensile mechanical properties due to a better adhesion with the matrix. Images of bCNF-based biocomposites demonstrated that bCNFs are good reinforcing agents as the fibers were dispersed within the starch film and embedded within the matrix. Roughness increased with CNF content and decreased with CNC content. Films with CNCs did not show bacterial growth for *Staphylococcus aureus* and *Escherichia coli*. This study offers a new theoretical basis since it demonstrates that different proportions of bleached or unbleached nanofibers and nanocrystals can improve the properties of starch films.

## 1. Introduction

There has been an increasing interest in generating and producing biodegradable plastics from natural polymers. Novel technologies developed with biodegradable polymer exploit the individual characteristics of polysaccharides, proteins, and lipids in a wide range of applications, from packaging and biomedical devices to the food field, due to their biocompatibility, non-toxicity, mechanical integrity, and environmentally friendly nature [1].

Starch is an abundant and natural polymer obtained mainly from plants. Due to its inherent properties, such as degradability, availability, low cost, and renewability, numerous starch-based biocomposites have been developed [2,3,4]. Starch is composed of D-glucose monomers bonded by α(1–4) linkages [5]. Two main starch structures are found in the form of linear amylose (20–30%) and branched amylopectin (70–80%) [6,7].

Although plasticized starch is a promising material, its highly hydrophilic character and poor mechanical properties hinder its usage compared to conventional polymers [8,9]. To improve the properties of starch, reinforcement with more resistant biodegradable polymers or by chemical techniques are valid approaches, such as polyacid lactic acid [10,11], cross-linking [9,12], and reinforcement with mineral particles, cellulose nanofibers (CNFs), or cellulose nanocrystals (CNCs) [13]. Huang L. et al. (2020) report composite films using cassava starch reinforced with modified and unmodified CNFs obtained from cassava residue. CNFs were treated with malic acid for esterification and silane as a cross-linking agent, KH-550. The use of CNFs, modified and unmodified, as reinforcement material on films improves mechanical, hydrophobic, and barrier properties. The results show that a concentration of 3 wt% of nanofiber is the optimal concentration of nanomaterial to enhance the properties of starch films [14].

Agave bagasse is a residual fibrous material that remains after extraction of the fermentable juice from *Agave tequilana* Weber var. Azul pineapple. About 40% of the total weight of consumed agave belongs to bagasse residue. According to the Tequila Regulatory Council, 523,600 tons of bagasse were obtained from *A. tequilana* in the tequila generation process during 2020 [15]. Excess bagasse creates a substantial environmental impact on agricultural waste management and disposal [16]. In the last decade, efforts have focused on exploiting *A. tequilana* bagasse. The high cellulose content of *A. tequilana* bagasse, close to 80% [17,18], allows the production of high-added-value by-products, such as CNCs [19,20,21] and CNFs [22,23,24].

CNCs and CNFs mainly differ in length and aspect ratio. CNCs typically display diameters between 2 and 20 nm and lengths of <500 nm [25], whereas CNFs’ size varies from 1 to 100 nm in diameter and 500 to 2000 nm in length [26]. Due to their inherent desirable mechanical properties, CNCs and CNFs have already been employed as reinforcement agents on different biocomposite films. On one hand, the lower aspect ratio of CNCs can be exploited for durable, resistant, and edible materials. For example, the reinforcement of starch films with CNCs from sugarcane bagasse improved the water resistance and water barrier properties [27], and the reinforcement with CNCs from mango seed shells improved the tensile properties and water vapor barrier [28]. Cellulose nanocrystals from cassava residues were modified with ester, using soybean oil, to obtain CNCs/starch films with improved polarity and hydrophobicity [29]. Starch films were reinforced with kaolin, observing improved thermal and barrier properties [30]. The effect of CNC reinforcement of suspensions containing residual salt on the mechanical properties of thermoplastic starch nanocomposites was evaluated, reporting that by reinforcing starch films with up to 5 wt% of CNCs, stiffness and strength are improved [31]. On the other hand, the excellent compatibility between CNFs and glycerol from thermoplastic starch matrix allows for better fiber dispersion and the preparation of superior biocomposites in terms of physical, chemical, and mechanical properties. For example, thermoplastic starch films reinforced with nanofibers from *Agave fourcroydes* showed increased tensile strength and elastic modulus and decreased water vapor and oxygen rate [32]. Much of the work in this area has the objective of finding possible applications in food packaging [33] and to propose new renewable single-use disposables [34], according to the mechanical, barrier, thermal, or antibacterial properties of each formulation or production process used.

Cellulose nanofibers and nanocrystals improve the mechanical properties of native starch-based biofilms. In the present work, thermoplastic starch films prepared by solvent casting method and reinforced with CNCs, bleached CNFs (bCNFs), and unbleached CNFs (ubCNFs)—obtained from *A. tequilana* bagasse—were prepared and investigated via scanning electron microscopy (SEM), differential scanning calorimetry (DSC), thermogravimetric analysis (TGA), and Fourier-transform infrared spectroscopy (FTIR). Solubility, water vapor permeability, rugosity, color analysis, electrical conductivity, antibacterial, and tensile tests were also performed. 

## 2. Materials and Methods

### 2.1. Materials

Bagasse from *A. tequilana* Weber var. Azul was obtained from a local tequila company, Mundo Agave. (Tequila, Mexico). Sodium hydroxide (NaOH, 97% purity) and sodium thiosulfate (0.1 N) were from Karal S.A. de C.V. (León, Mexico). Anthraquinone (97% purity), cupriethylenediamine (CED), potassium permanganate, sulfuric acid (97% purity), sodium chlorite (97% purity), hydrochloric acid (37% purity), and hydrogen peroxide (30% purity) were purchased from Merck (Darmstdt, Germany). Glycerol was obtained from Golden Bell. Magnesium sulfate was obtained from Productos Químicos Monterrey (Monterrey, Mexico). Native corn starch was purchased from IMSA, Industrializadora de Maíz S.A. de C.V. (Guadalajara, Mexico).

### 2.2. Preparation of Agave Alkaline Pulp

A total of 1 kg of bagasse from *A. tequilana* Weber var. Azul was washed and cooked in a reactor (Jayme type Digester, R25W/3 model) with a solution of 22% NaOH and 0.1% anthraquinone (5:1) at 160 °C for two hours. The resulting pulp was filtered through a disc refiner (20 mm), shredded, and purified using diaphragm purification equipment provided with slotted plates with a #40 disc [17].

The chemical composition of agave pulp was determined via the standard TAPPI methods. The Kappa number (KN) was determined via the standard TAPPI T 236 om-22 [35], as follows: A sample of pulp with a known moisture content was first dispersed in 800 mL of distilled water at 25 °C. Then, 100 mL of 0.1 N KMnO_4_ and 100 mL 4 N H_2_SO_4_ were added. The solution was allowed to react for 10 min. After that period, 20 mL of 1 N KI was added to stop the reaction. Immediately after mixing, titration with Na_2_S_2_O_3_ was performed using starch (a few drops) as the indicator. A blank test (without pulp) was carried out with the same procedure. Calculations were made using the formulas from the standard. The test was carried out in duplicate. Viscosity (µ) and polymerization degree (PD) were determined under the standard TAPPI T230 om-19 [36], using the following procedure: First, 0.15 g of dry bleached pulp was dispersed in 25 mL of water. Then, 25 mL of cupriethylenediamine (C_18_H_13_N_4_NaO_7_S) 0.167 M was added to the solution. The system was purged with nitrogen and shaken until complete dissolution of the pulp (15–30 min). The dissolved pulp was transferred to a viscometer and adjusted at the lower mark of its capillary. The viscometer was put in a bath at 25 ± 0.1 °C until the vessel reached that temperature. The solution was vacuumed to the capillary’s upper mark, and the travel time from one mark to the other was registered. The measurements were realized in triplicate. The viscosity was determined by applying the formula stated in the standard. α, β, and γ-cellulose were determined under the standard TAPPI T203 cm-22 [37]. For these tests (α, β, and γ-cellulose), 1.5 g of pulp (as if dried) was first mixed with 75 mL of 17.5% NaOH. When the pulp was well dispersed, another 25 mL of 17.5% NaOH solution was added. This solution was allowed to react for 30 min at 25 °C. After that period, 100 mL of distilled water was added to the solution and stirred for another 30 min. Then, the fluid was subjected to filtration, and around 100 mL of the filtrate was collected (after discarding the first 20 mL of the filtrate). For α-cellulose determination, 25 mL of the filtrate and 10 mL of 0.5 N K_2_Cr_2_O_7_ were mixed, then 50 mL of concentrated H_2_SO_4_ was carefully added. The solution was allowed to be hot for 15 min. Then, the solution was cooled down to room temperature using water. After that, titration was performed using ferroin (phenanthroline/ferrous sulfate) as an indicator (2–4 drops) and 0.1 N Fe(NH_4_)_2_(SO_4_)_2_ solution. Titration ended when the solution turned purple. For titration of the blank test, the filtrate was replaced by 12.5 mL of 17.5% NaOH and 12.5 mL of water. In the case of β- and γ-cellulose determination, 50 mL of the filtrate was mixed with 50 mL of 3 N H_2_SO_4_, and then the solution was heated to 70–80 °C for a few minutes. The resulting precipitate was removed from the solution by filtering the next day. The next step was to add 10 mL of 0.5 N K_2_Cr_2_O_7_ to 50 mL of the solution. Then, concentrated H_2_SO_4_ (90 mL) was added carefully. The solution was allowed to remain hot for 15 min. Titration was made using the same procedure described for α-cellulose. Titration of the blank test was carried out using, instead of the solution, 12.5 mL of 17.5% NaOH, 12.5 mL of water, and 25 mL of 3 N H_2_SO_4_. The corresponding calculations were made according to the standard. The tests were carried out in duplicate.

### 2.3. Chlorine-Free Pulp Bleaching

An amount of agave alkaline pulp (100 g of pulp as if dried) was subjected to a chlorine-free bleaching sequence according to Gallardo et al., 2019 [17]. The steps were as follows: (1) Oxygen stage (O), with 2.5% NaOH and 0.2% MgSO_4_, with a consistency of 10%. The pulp was mechanically stirred and transferred to a preheated reactor at 100 °C (Jayme type Digester). Oxygen was pumped into the reactor for 5 min, and the reaction was allowed to perform for 60 min at 100 °C. The treated pulp was then washed and centrifuged; (2) Chlorine dioxide first stage (D0), with chlorine dioxide at 25% KN (pH 2–3) in an isothermal bath at 60 °C for 60 min. The treated pulp was again washed and centrifuged; (3) Oxygen peroxide reinforced stage, with a 1 N NaOH and 2.4 N H_2_O_2_ solution in an isothermal bath at 70 °C for 60 min. The treated pulp was again washed and centrifuged; (4) In the chlorine dioxide final stage, the pulp was placed in a polyethylene bag with a 2.5% chlorine dioxide solution, with a consistency of 10% (pH 2–3), then the sample was heated in a thermal bath at 80 °C for 180 min. The treated pulp was washed and centrifuged; (5) Oxygen reinforced peroxide stage, during which the pulp was mixed with a 2.4 N H_2_O_2_ solution (1.5%) at a consistency of 10% and heated in a thermostatic bath at 80 °C for 240 min. The treated pulp was washed, centrifuged, and stored at 4 °C until further use.

#### Scanning Electron Microscopy (SEM)

Bleached and unbleached pulp fibers were examined using a TESCAN MIRA3 scanning microscope from Keyence (Osaka, Japan). All samples were coated with a thin layer of gold. Average diameters were obtained by measuring a hundred diameters per type of fiber.

### 2.4. Cellulose Nanofibrils Isolation and Characterization

The isolation of cellulose nanofibrils from the treated agave pulp was carried out using a colloid mill (Super Mascolloider Microprocessor, from Masuko Sangyo Co., Ltd., Kawaguchi, Japan). The pulp suspension, with a consistency of 2%, was filtered five times through two discs at 1500 rpm, with a distance between discs of 0.1 mm.

The obtained cellulose nanofibrils were analyzed with a Transmission Electron Microscope (JEOL JEM 1200EX-II from JEOL Ltd. Tokio, Japan) coupled with a Gatan CCD high-resolution camera (Orius SC1000B). The microfibers were previously diluted at 0.002 wt% in distilled water, sonicated for 10 min, and placed on a palladium grid.

### 2.5. Cellulose Nanocrystals Preparation

Cellulose nanocrystals (CNCs) were obtained from soluble-grade cellulose pulp (α-cellulose > 90%) from *A. tequilana* Weber Var. Azul bagasse [19]. Acid hydrolysis was carried out with a 65% sulfuric acid solution at 60 °C for 70 min. The hydrolysis product was then dialyzed at pH 5.5, replacing the water every 12 h. Next, the suspension was filtered through 2, 1.5, and 1 µm filters and stored at 4 °C until use. The residual concentration of sulfate groups on the CNC-S surface (mmol/kg) was measured by conductometric titration as depicted for CNCs from huizache wood [38].

### 2.6. Atomic Force Microscope (AFM)

The morphology of CNCs was characterized using Park Systems AFM NX10 (from Park Systems Corp, Suwon, South Korea). CNC dispersions (0.005% in water) were sonicated in an ultrasonic bath. Next, 10 mL of CNC dispersions was transferred to a metal microscope slide and air-dried. CNCs images were obtained via tapping mode in the air using Budget Sensors Tap300-G probe (Izgrev, Sofia, Bulgaria). AC160TS-R3 micro silicon tips, coated with aluminum from Oxford Instruments (Abingdon, UK), were used for image recording. The amplitude and height of the samples were obtained from 1 μm × 1 μm images and a resolution of 512/512 pixels/line. At least 30 CNCs were measured, and the AFM dimensions (L, H, and W) were obtained.

### 2.7. Starch and Biocomposites Films Preparation

**Figure 1:**
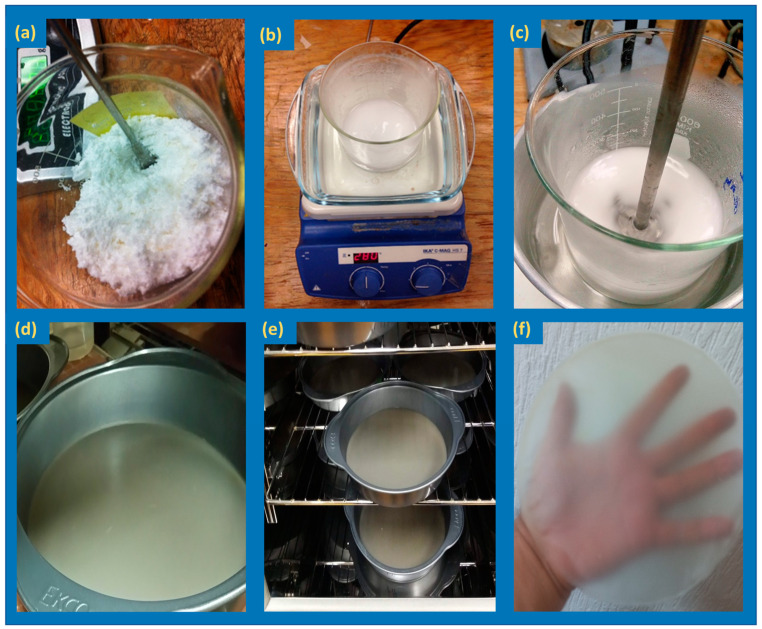
Preparation of films: (**a**) starch, (**b**) gelatinization, (**c**) incorporation of nanocelluloses, (**d**) deposition in the mold, (**e**) drying, (**f**) final film.

A film-forming solution was prepared by mixing native corn starch (5%) in 95% of deionized water and glycerol (30%), and different concentrations (0, 2.0, and 6.0 wt%, regarding the amount of starch) of CNCs, bleached CNFs (bCNFs), and unbleached CNFs (ubCNFs). The solution was kept under constant agitation at 80 °C for 30 min. The gelatinized dispersion was then sonicated (Branson M2800H) for 5 min to eliminate air bubbles. Next, the dispersion was poured on Teflon-covered metallic molds and placed in an air-circulating oven (LUZEREN DHG-9070A) at 30 °C, 53% RH for 15 h (Figure 1). The thicknesses of films were measured in five aleatory positions per film using a micrometer (Mitutoyo., Numata Japan, accuracy of 0.001 mm).

### 2.8. Characterization of Biocomposites Films 

#### 2.8.1. Color Analysis, Crystallinity, X-ray Diffraction and Electrical Conductivity

Color measurements of thermoplastic starch and CNC-, bCNF-, and ubCNF-composite films were taken at 10 random points with a USB2000 UV-Vis-NIR Spectrometer (OceanOptics). The results were expressed according to the CIELab scale (Commission International de I’Eclairage), where L denotes lightness (L: 0 = black and 100 = white) and a* and b* chromaticity: −a* (greenness) to +a* (redness) and −b* (blueness) to +b* (yellowness). Color differences were calculated as follows [39]:

∆E* = [(∆L*)^2^ + (∆a*)^2^ + (∆b*)^2^]^1⁄2^ (1)

where ΔL* = Li* − L* is the brightness difference, Δa* = ai* − a* is the red-green chromaticity difference, Δb* = bi* − b* is the yellow-blue chromaticity difference, and i is the reference value of each parameter.

The crystallinity changes were tracked by X-ray diffraction (XRD). Measurements were recorded using an Empyrean diffractometer (Malvern Panalytical, Worcetershire, United Kingdom) with Cu Kα radiation, operated at 40 kV and a current of 30 mA. Samples were scanned under 2θ range from 0 to 60°, in steps of 0.26° at 30 s/step. Samples of 25 × 40 × 0.127 ± 0.027 mm were dried at 30 °C for 60 min and placed on the sample holder with adhesive tape to keep the surface flat.

The crystallinity value of biocomposite films was determined according to Nara et al., 1978 [40], which is based on the relationship between the area of the crystalline zone and the total area under the diffraction pattern. Each point of the minimum peak intensity of the XRD pattern was joined by a smooth curve using Origin Pro 8.5 under a Gaussian model. The areas above and under the smooth curve correspond to the crystalline and amorphous regions. The crystallinity degree was calculated as a ratio between the area corresponding to the crystalline region and the total area under the curve.

Electrical conductivity was measured using a 6-½ digit precision multimeter (Tektronix DMM 4050, Beaverton, OR, USA). Fifty measurements in 5 different places on the films were carried out. The measuring distance was 1 inch.

#### 2.8.2. Solubility and Water Vapor Permeability 

Water vapor permeability (WVP) was determined gravimetrically using the ASTM E96/E96M-22a method [41]. Approximately 11 g of anhydrous silica gel was placed in each glass jar with an ornate plastic lid to establish dry conditions. The test area was 4.667 ×10^−4^ m^2^. Once the films were mounted, the entire set (bottle + lid + sample + silica) was weighed and placed in a Binder KBF Series climatic chamber for temperature control at 25 ± 1 °C and a relative humidity of 80% using a calcium chloride solution. The set was weighed every hour for the first 6 h and then every 24 h, with a precision balance of 0.0001 g readability. WVP was determined using the following equations:

WVP = (WVTR)/S(R_2_ − R_1_) (2)

WVTR = (G/t)/A (3)

where G is the change in weight (g), t is the time (h), A is the test area (m^2^), S is the saturation vapor pressure of water at the test temperature (Pa), R_1_ is the relative humidity at the source expressed as a fraction (0.0), and R_2_ is the relative humidity at the steam sink expressed as a fraction (0.8).

For solubility determination, 3 square test pieces (2 × 2 cm) were cut from each film and dried at 110 °C for 24 h in a ventilated drying oven. After drying, the films were weighed on a balance with a precision of ±0.0001 g to determine the initial dry weight (W_0_, expressed as dry matter). The film samples were placed in 50 mL of distilled water, covered, and stored at 25 °C for 24 h. Finally, the pieces of film were removed and dried at 110 °C to a constant weight (W_f_, undissolved dried film). The water solubility (W_S_ %) of the films was calculated using the following equation:

W_S_ (%) = ((W_0_ − W_f_)/W_0_) × 100. (4)

#### 2.8.3. Fourier-Transform Infrared Spectroscopy (FTIR-ATR)

Fourier-transform infrared (FTIR) spectroscopy measurements (in a L280 PerkinElmer instrument from PerkinElmer, Walham, MA, USA) were performed on films and cellulosic fibers. Spectra were collected within the 500–4000 cm^−1^ range (resolution of 4 cm^−1^). Deconvolution of peaks from the spectra was conducted to obtain the peak area ratios at different wavelengths. The analysis was performed for two specimens per sample. The deconvolution of peaks was obtained using the first derivative method in the OriginPro 2021 software from OriginLab Corporation (Northampton, MA, USA).

#### 2.8.4. Differential Scanning Calorimetry (DSC) and Thermogravimetric Analysis (TGA)

Differential scanning calorimetry was used to characterize the TPS and their biocomposites thermally. DSC thermograms were recorded on a TA Instruments calorimeter model Q-100 (New Castle, DE, USA). A dynamic heating program from −20 to 150 °C was followed at a heating rate of 10 °C/min, with a nitrogen flow rate of 50 mL/min to keep an inert atmosphere in the sample cell. Thermogravimetric analysis of TPS and biocomposites was performed on a TA Instruments thermobalance model TGA5000 Discovery. The results were recorded by heating the samples from 50 to 600 °C at 10 °C/min under a nitrogen atmosphere, generated by a flow rate of 25 mL/min.

#### 2.8.5. Mechanical Properties

Tensile tests were performed under the ASTM D882-18 norm [42] in a universal mechanical testing machine (INSTRON, Norwood, MA, USA) at 25 mm/min. Sample dimensions of 10 mm wide, 170 mm long, and 0.217 mm thick were first incubated at 50% relative humidity for 48 h.

#### 2.8.6. Scanning Electron Microscope (SEM) and Rugosity

Starch, CNC, bCNF, and ubCNF biocomposite films were examined by SEM using a TESCAN MIRA3 scanning microscope. Film samples were coated with gold and observed using an accelerating voltage of 10 kV. The rugosity was measured using contact profilometry Veeco Dektak 150 Surface Profiler from Veeco Instruments, (Tucson, ZA, USA).

#### 2.8.7. Antibacterial Properties

In order to establish the antibacterial behavior of the synthesized films, the disk-diffusion method was used based on the methodology reported by M. Mudassir [43], using two bacterial models, *Staphylococcus aureus* and *Escherichia coli*. Both bacteria were inoculated with 125 μL of bacteria with an optical density of 0.4 at 600 nm of absorbance for every 25 mL of Mueller–Hinton solid medium. This medium was deposited in Petri dishes, allowing them to solidify at room temperature under aseptic conditions. Subsequently, 6 mm discs of each film were placed, and kanamycin was placed as a control at a concentration of 25 μg/mL. The plates were left incubating for a period of 24 h at 37 °C. 

To perform the antimicrobial susceptibility assay, a solution of Tripticase Soy Agar (TSA) was sterilized at 121 °C and 4 psi for 15 min. A total of 25 mL of Agar solution was placed in a Petri dish, and when the solution reached room temperature, it was inoculated by streaks with the strains of *Staphylococcus aureus* and *Serratia marcescens*. Subsequently, 6 mm diameter discs, each of the biocomposites, were placed in the Petri dishes and incubated at 37 °C for 16 hrs. In addition, a positive control was also developed, which consisted of a sterilized Whatman filter paper disc previously immersed in an ampicillin solution (100 µg/mL) and compared with discs gripped in solved biofilms. 

The minimum inhibitory concentration was determined using the resazurin-based microdilution method [44,45]. The samples to be analyzed consisted of 5 mg films solved in 1 mL of LB medium and sterilized DMSO at 2% (*v*/*v*). A total of 300 µL of each sample was placed in triplicate in the second column of a 96-well plate. Serial dilutions were made from this sample by adding 150 µL of previous solution and 150 µL of fresh medium (3 to 9 columns). Then, 15 µL of the culture solution was added to 1 × 10^6^ CFU/mL of the *S. aureus* bacteria to each dilution. The incubation process was carried out at 37 °C for 18 h. Finally, 30 µL of 0.015% resazurin solution was added to each dilution and incubated at 37 °C for one hour. The first and last colon correspond to the negative and positive control, respectively. The MIC is considered the lower concentration where there is no color change. 

### 2.9. Statistical Analysis

All samples were analyzed at least in duplicate, and the results were reported as the mean. Statistical analysis was performed using OriginLab with two-way ANOVA. The two factors, A and B, were composite concentration (2.0 and 6.0 wt%) and composite type (ubCNF, bCNF, CNC), respectively. The *p* values are reported as pA, pB, and pAB to determine if A and B are significantly different and if the interaction between A and B is significant. The significance level used was 0.05.

## 3. Results and Discussion

### 3.1. Preparation of Agave Pulp

A total yield of 48% was obtained after the cooking process of agave bagasse. The obtained pulp had an average Kappa number of 21.2 ± 1.32. The reported Kappa number in this work is significantly lower than that from other authors, which can be attributed to the effect of anthraquinone. For example, Idarraga et al. carried out soda pulping of *A. tequilana* Weber var. Azul with 20 wt% NaOH at 175 °C for 2 h [46]. The reported Kappa number was 60, with a mass yield of 57.9%. Anthraquinone can cause oxidation of the terminal reducing group of carbohydrates, generating stability in terms of terminal depolymerization reactions [47]. On the other hand, anthraquinone causes an acceleration of hydrolysis reactions on lignin, forming fragments of lignin of lower molecular weight, which is reflected in an increase in the degree of delignification [48].

**Figure 2:**
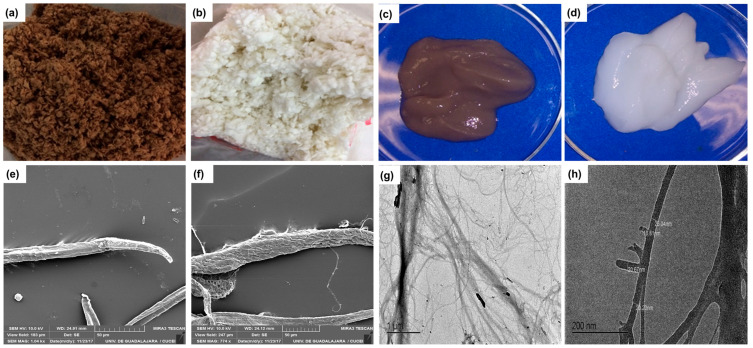
Photographs of (**a**) unbleached and (**b**) bleached agave bagasse pulp, (**c**) unbleached and (**d**) bleached pulp obtained after the fibrillation process. SEM micrographs of (**e**) bleached, (**f**) unbleached A. tequilana bagasse cellulosic pulp after the cooking process. (**g**) and (**h**) TEM micrographs of bleached agave nanofibers.

Figure 2a,b shows the pulps obtained before and after bleaching. After the bleaching sequence, an average whiteness of 85.7 was obtained. The α-cellulose analysis values of 79.4 ± 3% and 94.2 ± 2% were obtained before and after the bleaching process.

After the fibrillation process, a viscous matter was obtained (Figure 2c,d). The increase in apparent viscosity was due to the formation of a stronger network between the cellulose fibrils with an increasing degree of fibrillation [49]. In addition, the heat generated during the fibrillation process evaporated the water increasing the consistency of the microcellulose viscosity [50]. The fiber size reduction manifested the effect of the mechanical shear.

### 3.2. Characterization of Agave Fibers 

Figure 2 shows the SEM images of unbleached and bleached agave fibers, where pits can be observed. After the bleaching process (Figure 2e,f), cleaner fibers, individually and in aggregation, were obtained due to lignin removal. The diameters of unbleached and bleached fibers were 23.81 µm ± 6.85 and 22.3 µm ± 5.24. Mechanical shearing is not a completely controlled process that occurs at a relatively low temperature (below 60 °C), which is not high enough to soften the lignin, which hinders the internal fibrillation, thus producing a heterogenous microfiber size [51]. The fibers were highly hydrated, which is attributed to the exposure of –OH groups of cellulose after mechanical fibrillation.

As micro and nanofibers are usually entangled due to fibrillation, TEM observations are performed to determine the nanometric size. Figure 2g,h shows the cellulosic fibers, obtained after the fibrillation process, in which highly interlaced ultrafine and structural fibrils were observed. Cellulose fibers showed an interconnected network structure containing various individual nanofibers with a diameter of less than 100 nm and a length greater than 1 µm [52].

The degree of polymerization (DP) of cellulose fibrils is a valuable parameter to determine the properties of cellulose-reinforced materials, as the DP strongly influences the final mechanical properties. The DP decreased from 690 for fibrillated and unbleached fibers to 491 for non-fibrillated and bleached fibers. Mechanical grinding physically destroys cellulose crystals and breaks cellulose chains, which can cause a decrease in DP [50]. However, the mechanical fibrillation process caused a more significant reduction in the value of the degree of polymerization of the fibrillated pulps containing lignin (410 for fibrillated unbleached fiber) compared to the bleached pulp (435 for fibrillated and bleached fiber). The presence of hemicelluloses and lignin favor mechanical fibrillation [51,53]. Due to their hydrophilic nature, hemicelluloses aid the swelling of fibers, whereas lignin, due to its hydrophobic nature, aids fibrillation since it counteracts the recombination reactions between cellulose molecules. Therefore, the presence of both components improved fiber delamination in the aqueous suspension [54,55].

### 3.3. CNCs Isolation and Characterization

**Figure 3:**
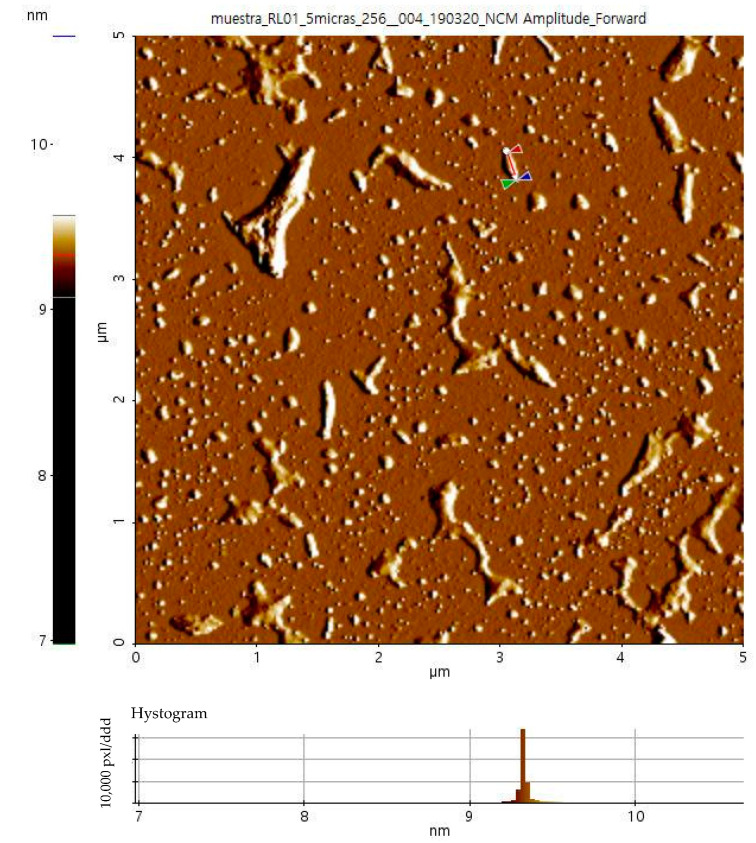
AFM 2D image and histogram of CNCs.

The total acid group content of CNCs quantified was 160 µmol/g. The average size of CNCs was ~300 nm. Figure 3 shows the AFM 3D projection and 2D image height histogram of CNC marked in Figure 3 as a reference. While in Figure 3 it is observed the presence of long, wide, and heterogeneous crystals, with an average length 280 ± 97 nm, and wide of 78 ± 15 nm; in Figure 3, the histogram depicts the height of nanocrystal marked in Figure 3, which is 8.7 nm. These results are consistent with the length and width values also observed with CNCs from *A. tequilana* obtained with 60 wt% sulfuric acid (length of 137–404 nm, width of 8.7–9.3 nm) [19].

### 3.4. Starch and Biocomposites Films Preparation and Characterization

**Figure 4:**
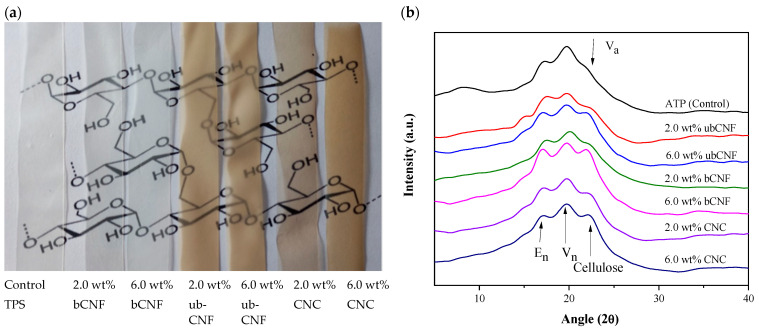
(**a**) Visual appearance of thermoplastic starch, bCNF, ubCNF, and CNC biocomposite films. (**b**) XRD spectra of thermoplastic starch, bCNF, ubCNF, and CNC biocomposite films.

Film biocomposites of approximately 0.2 mm thick and 170 mm in diameter were obtained using the solvent casting method. The average thicknesses of the films are shown in Table 1. Thermoplastic starch-based biocomposite films showed a homogeneous appearance without agglomerates, good dimensional stability, and no fractures. The microstructure and thickness of the films affect the optical properties of the films, which are greatly influenced by the internal and surface heterogeneity of the matrix [56]. The visual inspection of films reinforced with bCNFs demonstrated a slight color change to brown, whereas films reinforced with ubCNFs and with CNCs showed an initial light-brown coloration, with increasing color intensity and with increasing CNC concentration (Figure 4a). In all cases, transparency was maintained. Table 1 shows the resulting film biocomposites’ L*, a*, and b* parameters. In all cases, the brightness decreased with the addition of bCNFs, ubCNFs, and CNCs. The brightness depends on the surface morphology reached during the drying phase of the film, and, in general, smoother surfaces give higher brilliance values [57]. Similarly, a* chromaticity values were observed for films reinforced with 2.0 wt% and 6.0 wt% of unbleached CNFs, whereas a* increased towards the red for bCNF and CNC composites, with increasing concentrations of bCNFs and CNCs. Similar results were observed for b*, in which an increase was observed in all cases towards yellow.

The color difference (ΔE*) of the films increased with increasing nanocellulose concentrations, being higher for ubCNF-based films (34.05 ± 1.39, 6.0 wt% of ubCNFs), followed by the CNC films (22.07 ± 1.02, 6.0 wt% of CNCs) and the bCNF films (13.66 ± 0.44, 6.0 wt% of bCNFs). Similar results were also found for biocomposite films of native potato (L = 95.70, a = −5.14 and b = 7.38) [58], quinoa starch (L = 92.86, a = −0.87 and b = 1.96) [59], and tapioca starch (L = 85.42, a = −1.08 and b = 5.02) [60].

Figure 4b shows the diffraction peaks (2θ) E_h_, V_h_, and V_a_ (17°, 19.6°, and 21.8°, respectively) of thermoplastic starch, in which the recrystallization of amylose chains is observed [61,62]. V_h_ has a six-fold left-handed helix in an orthorhombic unit cell. In contrast, the V_a_ lattice, also indexed as an orthorhombic unit cell, has more contracted amylose helices and contains less water than the V_h_ lattice. Conversely, E_h_ has been described as a seven-fold single helix in a hexagonal unit cell [62]. In the spectra of the biocomposite films, two peaks at ~15.65° and 22.5° correspond to the cellulose naturally present in starch films. The peak corresponding to V_a_ is hardly noticeable as it appears in very close contact with the cellulose peak and might have been overlapped [63,64,65]. Incorporating CNCs leads to the amorphous halo’s growth and to the decrease in peak intensities induced by the processing. This might be explained by interactions between the nanocrystals and the starch macromolecules or by the hindering of glycerol of the crystalline arrangement of the amylose–glycerol complex [65]. In the spectra of the biocomposite films, two peaks at ~15.65° and 22.5° correspond to the cellulose naturally present in starch films and increase as the ubCNF, bCNF, and CNC content increases on the films [66].

Some authors have reported degrees of crystallinity values of thermoplastic starch between 10% and 30% depending on the amylose content, the arrangement of the internal structure, and the processing method [67]. The crystallinity values shown in Table 1 showed an increasing trend in the cellulose concentration of CNCs, bCNFs, and ubCNFs in the biocomposite films, which is directly related to the physical and mechanical properties of the materials.

The electrical conductivity of the films is reported in Table 1, and it is evident that it slowly increased according to the following order of samples: TPS < bCNF < ubCNF < CNC composites, which could be attributed to sulfate groups of cellulose nanocrystal and lignin on CNFs, even though the values are similar, indicating that starch film conductivity is not affected by the composite. 

### 3.5. Solubility and Water Vapor Permeability

One of the main disadvantages of starches is their highly hydrophilic character. The hydroxyl groups present in the starch structure make it a polar substance that easily attracts water molecules from the environment. The influence of humidity on the properties of TPS films can become significant. The interaction with water drastically changes the mechanical properties of a thermoplastic starch-based material because it can weaken the interaction between starch molecules and make molecular movement more flexible, facilitating the retrogradation process [68].

### 3.6. Fourier-Transform Infrared Spectroscopy (FTIR)

**Figure 5:**
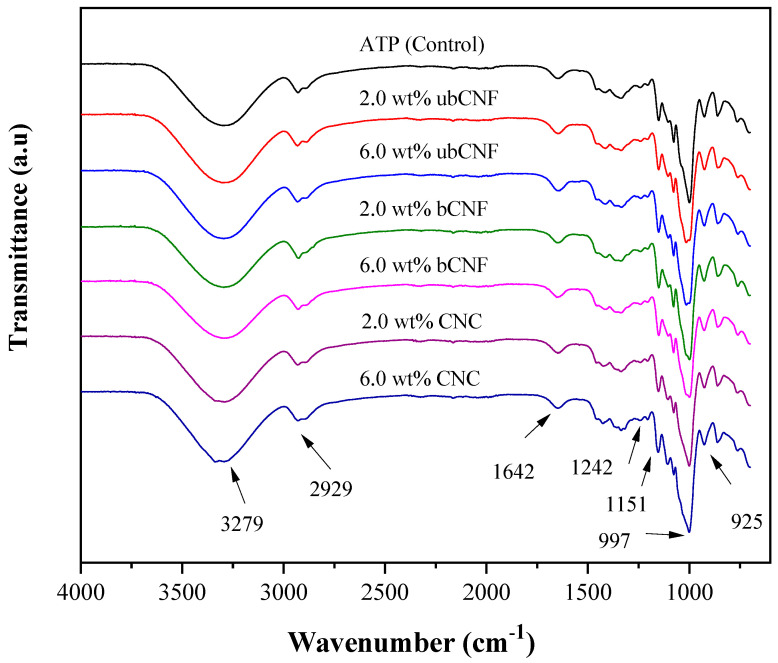
FTIR spectra comparison of thermoplastic starch: CNC, bNCF, and ubCNF biocomposite films.

Figure 5 shows the FTIR spectra of the TPS and composite films. Characteristic bands of cellulosic materials were observed in all samples. A broad band between 3000 and 3500 cm^−1^ corresponded to the stretching vibrations of the -OH groups and hydrogen bonds. Bands between 2880 and 2950 cm^−1^ were attributed to the stretching vibrations of -CH from the starch chains [69,70]. A small signal was observed at 1643 cm^−1^, which corresponded to water, while peaks located between 1200 and 1450 cm^−1^ and 1030 and 1160 cm^−1^ were attributed to the bending and stretching vibrations of -CH, C-C, -OH, C-OH, and C-O-C bonds [2,17,71,72].

All spectra display the same bands since they have the same functional groups and bonds. In order to analyze the influence of the nano-reinforcements, peak area ratios were obtained from the spectra (shown in Table 2). Deconvolution was conducted to obtain peak areas at different wavenumbers.

The bands at 763, 861, 925, 1077, and 3280 cm^−1^ corresponded to the starch [73], with no interference from the nanoparticles. Peak area ratios of 1077 cm^−1^/925 cm^−1^ and 861 cm^−1^/925 cm^−1^ corresponded to bond vibrations of C-OH (1077 cm^−1^), deformation vibrations of C1(H) and CH_2_ (861 cm^−1^), and the vibration mode of the α(1–4) glycosidic backbone (925 cm^−1^) (C-O-C) [70]. The AREA_1077_/AREA_925_ ratio for the control was 0.74 ± 0.01, and for the composite films, the ratio was 0.66 ± 0.01. Regarding the AREA_861_/AREA_925_ ratio, the control sample was 0.94 ± 0.04 and 0.89 ± 0.02 for the composite films. The results in Table 2 confirm that both bands (1077 cm^−1^ and 861 cm^−1^) belonged to TPS and the nanoparticles did not impact them. In both cases, the ratio was higher for the control sample. Moreover, the relationship between the strain vibration of C1(H) and CH_2_, and the vibration of the α(1–4) glycosidic backbone (AREA_2929_/AREA_925_) [74,75,76,77,78] remained practically invariant in both the presence and absence of nanoparticles. Another band with the same behavior is the one located at 3280 cm^−1^. 

To discuss the effect of the cellulosic nano-reinforcements on TPS films, an internal correction is made using the band at 925 cm^−1^, which is not impacted by the nanoparticles. AREA_λ_/AREA_925_ ratios for values of λ of 1573, 1020, 1341, and 1415 cm^−1^ showed that ratios decreased according to the following order of samples: ubCNF > bCNF > CNC composites. Furthermore, in most cases, it is the samples with ubCNFs that exhibited a higher value than the control sample, while the pieces with CNCs had a deal close to that of the control sample. This effect could be due to the area representing the total band. It is essential to mention that absorbance ratios were not calculated since they can be affected by the smoothing process, baseline, etc. For this reason, analysis of areas seems to be a better approach to evaluate the effect of the variables. The band observed at 1573 cm^−1^ was attributed to the C=C vibrations of aromatic rings in ligins and extractable [75]. The decrease in the AREA_1573_/AREA_925_ ratio in composites containing bleached and hydrolyzed reinforcements indicated the removal of the aromatic compounds, which most likely occurred during the bleaching step or the hydrolysis step. The band at 1020 cm^−1^, attributed to the amorphous part and the deformation vibration of C-O of cellulose [70], exhibits AREA_1020_/AREA_925_ ratios decreasing in the order of ubCNF composite > bCNF composite > CNC composite, indicating the presence of more amorphous regions in ubCNF-based composites, independently of concentrations. The same occurs with the bands at 1341 cm^−1^ and 1415 cm^−1^, which were attributed to the bending and vibration bonds of CH_2_ from starch and the C-O-O from cellulose [73], showing a slight decrease when the concentration of the nanoparticles increased from 2.0 to 6.0 wt%.

Moreover, for the band at 997 cm^−1^, related to the starch’s single-helix crystalline structure [73], the opposite occurred, presenting a higher value for CNC composites and a lower value for ubCNF composites (CNC composites > bCNF composites > ubCNF composites), indicating that this band is directly related to the crystallinity of the material, which can also be observed in the band at 1357 cm^−1^. The band at 1041 cm^−1^ is also directly related to the crystallinity of cellulose [73], and it can be observed that it increases from bCNF to CNC composites. Also, the AREA_1041_/AREA_925_ ratio indicated the elimination of amorphous material during hydrolysis, which increased the proportion of crystalline material in the film.

On the other hand, the presence of S=O groups can be detected via the band at 1206 cm^−1^, in which the areas ratio values for bCNF-based composites were 0.25 ± 0.01 and 0.26 ± 0.008 for 2 and 6 wt%. However, when CNCs were used as the reinforcing material, these ratios were 0.30 ± 0.002, regardless of concentration. 

It has been reported that absorbance and area ratios can provide information regarding the amorphous and crystalline structure [75]. The higher the value of the ratio, the higher the amorphous proportion of the material, or the lower the value of the ratio, the higher the crystalline proportion. In this context, the AREA_1020_/AREA_1077_ ratio was 1.21 ± 0.05 for the control film and then decreased according to the following order: ubCNF composites > bCNF composites > CNC composites. As the material’s crystalline proportion increased, the ratio’s value diminished.

### 3.7. Differential Scanning Calorimetry (DSC) and Thermogravimetric Analysis (TGA)

**Figure 6:**
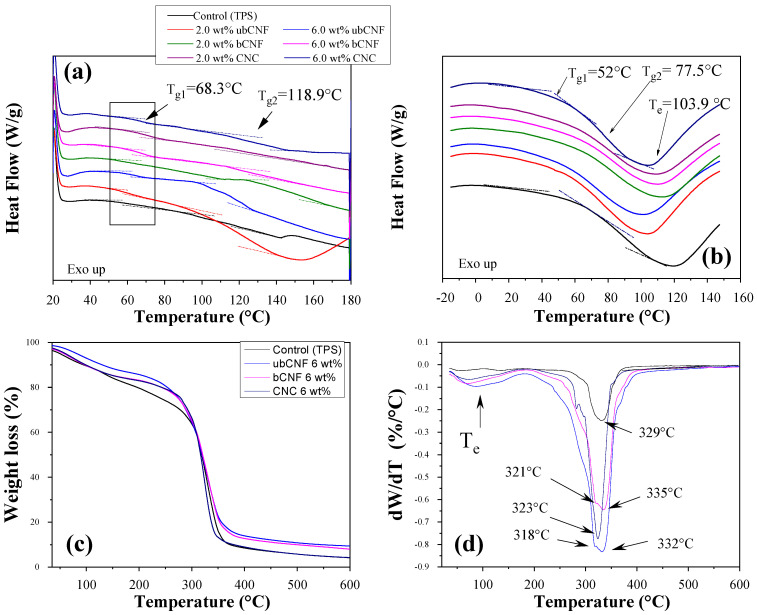
DSC thermograms of thermoplastic starch, CNC, bNCF, and ubCNF biocomposites films: (**a**) dried films and (**b**) non−dried films. (**c**) Thermogravimetric and (**d**) derivative thermogravimetric curves of thermoplastic starch biocomposites films.

For DSC measurements, all samples were placed in a vacuum oven at 40 °C for 48 h to remove moisture. Measurements were performed on samples before the dry process. Two transitions were detected in the TPS and their composites. Thermograms are shown in Figure 6a,b for dried and non-dried samples. In Figure 6a, that the control sample (TPS) shows that the first transition is located at 67 °C and the second is at 118.7 °C. The first transition increases with the concentration in ubCNF and bCNF biocomposites, and the values are reported in Table 3. These temperatures could be attributed to the glass transition temperature, and the T_g_ values coincided with those reported by other authors for TPS/cellulose films [79,80]. The second transition range appeared between 115 and 122 °C. Some authors have related this temperature with moisture loss, in addition to films of TPS and microcrystalline cellulose (MCC) obtained by the hot-pressing method with T_g_ from 115 to 126 °C [81]. Figure 6b shows a thermogram for TPS film, non-dried, with 6.0 wt% of CNCs. Comparing the results with those obtained for dried samples, the T_g_ diminishes from 88.3 to 52 °C because of the moisture content [82,83]. The second transition goes from 118.9 to 77.5 °C, while the peak of water evaporation is located at 103.9 °C. The two transition temperatures could be attributed to the starch retrogradation process during heating to form films. All samples exhibit the same behavior and both temperatures diminish with the moisture of the films. 

Moreover, TGA characterization was performed to study the thermal stability of the composites. The TGA curves of the synthesized TPS and the TPS with 6.0 wt% of ubCNFs, bCNFs, and CNCs are shown in Figure 6c. The derivative of the mass loss per unit temperature as a function of temperature is shown in Figure 6d. It is observed that the first weight loss occurred at ~100 ◦C in all samples, inferring the hydrophilic nature of the cellulose fibers. The initial and final degradation temperatures are shown in Table 3 (ranging between 250 and 450 °C). The onset of the degradation temperature diminishes in the order TPS < ubCNF composites < bCNF composites < CNC composites. On the other hand, from the derivative curve (Figure 6b) for TPS and TPS with 6.0 wt% of CNCs, a single peak at 329 and 323 °C for each sample was observed. However, for composites with ubCNFs and bCNFs, there is a shoulder in the peak, which could be attributed to the amorphous material of cellulose present in the nanofibers and the absence of CNCs. The ash content at 600 °C of the biofilms is shown in Table 3 and is more considerable for ubCNF and bCNF biofilms than for TPS and CNC biofilms.

### 3.8. Mechanical Properties

**Figure 7:**
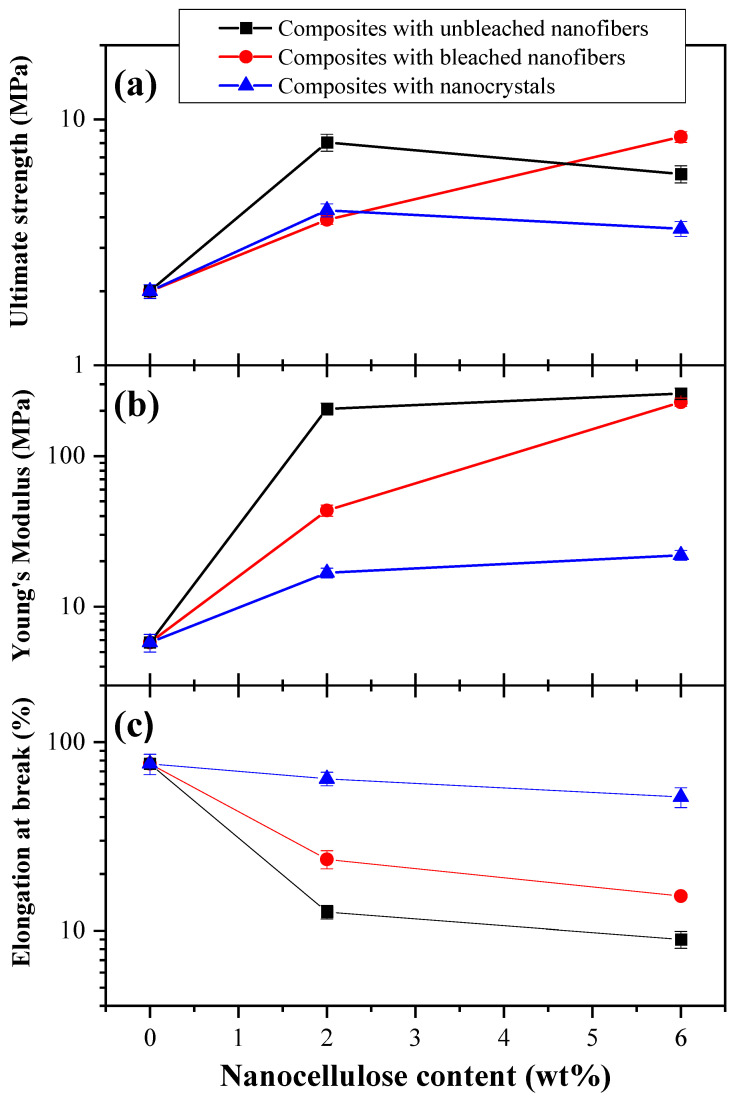
Mechanical properties of CNCs-, bCNFs- and ubCNFs-based composites. (**a**) Tensile strength, with *p* values of 0.448, 0.017, and 0.032 for A, B, and A and B interactions, respectively. (**b**) Young’s modulus, with *p* values of 0.002, 4 × 10^−6^, and 4 × 10^−6^ for A, B, and A and B interactions, respectively. (**c**) Elongation at break with *p* values of 4 × 10^−4^, 1 × 10^−11^ and 4 × 10^−11^ for A, B, and A and B interactions, respectively. (A and B factors: concentration and type of composite).

Figure 7 shows the results of the tensile strength (*T*), Young’s tensile modulus (*Y*), and elongation at break (%ε) for the obtained biocomposite films. The *T* value recorded for thermoplastic starch was 2.0 MPa. Overall, the addition of CNCs and CNFs increased *T*, *Y* and decreased %ε values, indicating good dispersity of the nanoparticles throughout the matrix [27]. This effect was more notorious for ubCNF composites, which might be explained by its highest aspect ratio, which has been linked to a better mechanical reinforcement capacity [27,28], and by the presence of lignin and hemicelluloses in the composition of the microfibers, which also confers mechanical resistance [71]. The lignin on biofilms was corroborated by FTIR-ATR at 1573 cm^−1^.

The *T* values recorded were 4.27 MPa and 3.59 MPa for 2.0 wt% and 6.0 wt% CNCs, respectively, 3.9 MPa and 8.05 MPa for 2.0 wt% and 6.0 wt% bCNFs, respectively, and 8.48 and 10.16 MPa for 2.0 wt% and 6.0 wt% ubCNFs, respectively. *Y* values for CNC composites remained almost unaltered with increasing CNCs concentrations (from 22.6 MPa to 27.7 MPa for 2.0 wt% and 6.0 wt% CNCs, respectively). In contrast, a greater increase in *Y* was observed for bCNF composites, at 43.51 MPa and 227.8 MPa for 2.0 wt% and 6.0 wt%, respectively. ubCNF-based composites gave the highest *Y* values, specifically 205.6 MPa and 277.3 MPa for 2.0 wt% and 6.0 wt%, respectively. These results agree with previous results reported in the literature [84,85,86,87]. The %ε values were dramatically affected by the type of nanocellulose used as reinforcing agent as the decrease in %e values was 5.5-fold for ubCNF composites compared to CNC composites. The contraction of the starch chains and the good distribution of CNFs in the matrix contributed to forming a more compact network with stronger chemical interactions, which overall reduced the material deformation when subjected to mechanical stresses. Similar results have also been reported in the literature [71,87,88].

Based on the results from the XRD and FTIR characterizations, it is clear that CNC-based composites were more crystalline than those containing ubCNFs and bCNFs. This was most likely due to the highly crystalline nature of the CNCs. Tensile strength values of CNC composites were lower than those from ubCNF and bCNF composites. This could be attributed to the higher crystallinity of the CNC composites, which can make them more brittle than others.

### 3.9. Morphology of Thermoplastic Starch and Biocomposite Films

**Figure 8:**
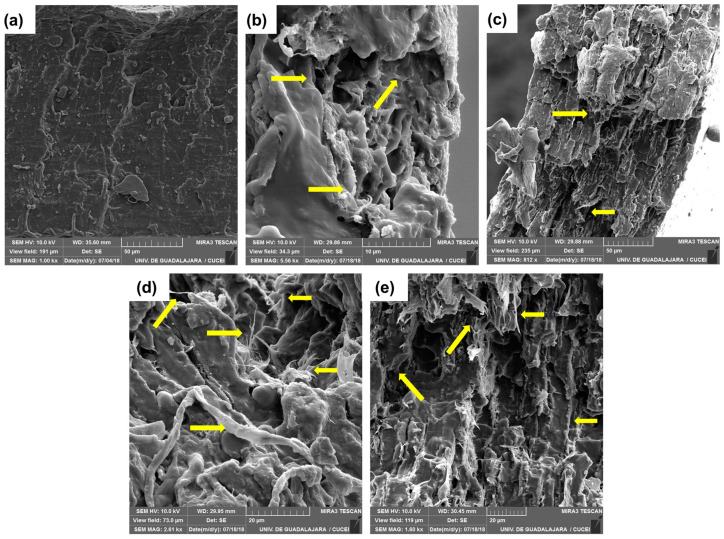
SEM micrographs of fractured (**a**) thermoplastic starch, (**b**) 2.0 wt% and (**c**) 6.0 wt% bCNF biocomposites, (**d**) 2.0 wt% and (**e**) 6.0 wt% ubCNF biocomposites. The arrows point to the microfibers embedded in the plasticized starch matrix.

Scanning electron images were obtained from the fracture zone of the tension test specimens (Figure 8). After plasticizing, thermoplastic starch specimens showed a smooth surface without starch granules (Figure 8a). Images of bCNF indicated good dispersion within the starch film as some microfibers were embedded in the matrix, thus demonstrating that bCNFs are good reinforcing agents. A rougher surface was observed for micrographs of 6.0 wt% bCNFs (Figure 8b), whereas a more porous structure was obtained for 2.0 wt% of bCNFs (Figure 8c). Similar morphologies were also observed for 2.0 wt% and 6.0 wt% ubCNFs. Small nanothreads, as defined by García et al., 2011 [89], or nanometric fibrillar structures could be observed for specimens with 6.0 wt% of bCNFs and ubCNFs (Figure 8d,e). These nanothreads are due to glycerol, which interacts with CNF, forming fibrillar clusters of CNF and glycerol. 

Table 3 shows the roughness results. An increase was observed in the presence of bleached and unbleached nanofibers and a decrease in the presence of nanocrystals. The addition of microfibrillated cellulose (MFC) increased the surface roughness and polarity of the starch/MFC materials developed [90]. Velásquez-Castillo et al. (2023) reported that in starch films reinforced with 5.0 and 7.5 wt% quinoa starch nanocrystals [91], roughness increased with the presence of nanocellulose. It was reported that the greater the thickness and surface roughness of starch-based films, the greater the contact area between the films and water, increasing the solubility of starch-based films [92].

### 3.10. Antimicrobial Properties

*Escherichia coli* is the most important foodborne pathogen. It is transmitted orally or fecally and should not be present in food under any circumstance. *E. coli* is an indicator bacterium in food safety and hygiene [93].

**Figure 9:**
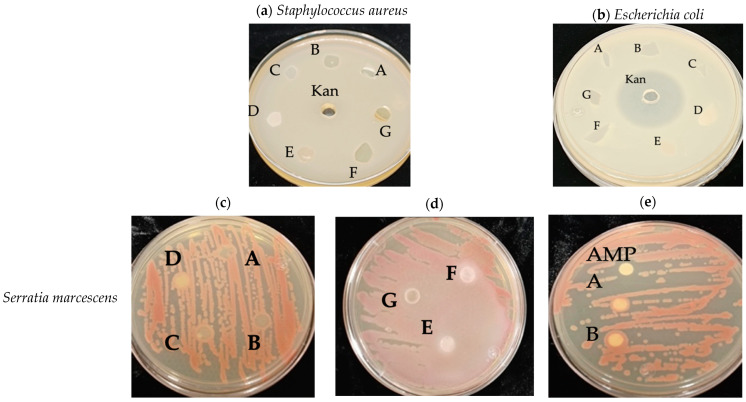

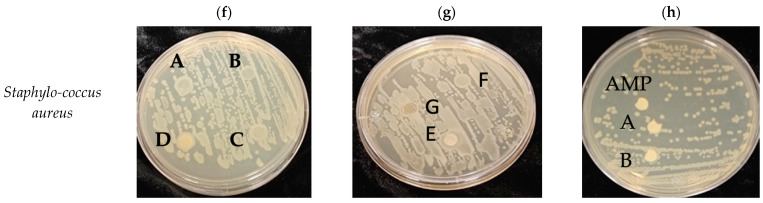
Antibacterial behavior of the synthesized films with (**a**) *Staphylococcus aureus* and (**b**) *Escherichia coli*. Antimicrobial susceptibility results as a function of the type of bacteria for biocomposite discs (**c**,**d**,**f**,**g**) and Whatman filter paper immersed in ampicillin and solved biofilms (**e**,**h**). A: TPS, B: 2.0 wt% bCNF, C: 6 2.0 wt% bCNF, D: 2.0 wt% ubCNF, E: 6.0 wt% ubCNF, F: 2.0 wt% CNC, G: 6.0 wt% bCNF, Kan: Kanamycin, AMP: ampicillin.

The results of the antibacterial tests show that the samples in which the bacterial growth in the material was not observed were 2.0 wt% y 6.0 wt% CNCs biocomposites for both the *S. aureus* and *E. coli* strains (Figure 9a,b). This inhibition could be observed in the upper and lower part of the plate; unlike the rest of the materials, they allow bacterial growth in them. The inhibitory halo for kanamycin control is observed in Figure 9b. Starch-based composite antibacterial films were successfully prepared by casting nanocellulose as the reinforcing material and polyhexamethylene biguanide (PHMB) as the antibacterial agent [94]. 

Figure 9 also shows the pictures of the antimicrobial susceptibility assay with *Serratia marcescens* (Figure 9c,d) and *Staphylococcus aureus* (Figure 9f,g) using biocomposite discs. In both cases, the absence of the inhibitory halo in all the samples is remarkable; instead, bacterial growth around the discs is denoted as a pink or white circumference in the disc for the two bacteria types. Nevertheless, up and down, there is no bacterial growth on the discs for all sample solutions and two types of bacteria. Furthermore, the positive control with ampicillin, as shown in Figure 9e,h, inhibited bacterial development, which was not observed in the Whatman filter paper discs immersed in solved samples (TPS and 2.0 wt% bCNF films). 

Only the solved films (TPS, 2.0 wt% bleached CNF, 2.0 and 6.0 wt% CNC biocomposites) were analyzed to determine the minimum inhibitory concentration. In Figure S1, the 96-well plate is shown; the negative control has a blue color, while the negative color has an amber color. It can be observed that the control sample (TPS), 2 wt% bCNF, and 2 wt% CNC samples did not exhibit an inhibitory bacterial growth. In contrast, the 6 wt% CNC biocomposite film had an MIC of 5 mg/mL. 

### 3.11. Potential Application of Film Composites

The films obtained can be joined by applying heat with electrical resistance, generating a good seal at the union. Heat sealing is another essential property for many packaging applications that hinders the practicality of simple starch film formulations [95]. Based on the properties that the films presented, such as flexibility, transparency, and homogeneity, it is suggested that they can be used as packaging for food and other materials containing low amounts of water. For films containing nanofibers, the use of a bactericide is recommended.

## 4. Conclusions

Currently, it is essential to promote the circular economy, which involves sharing, renting, reusing, repairing, renewing, and recycling existing materials and products as much as possible to create added-value products so that the life cycle is extended. This implies reducing waste to a minimum. The increase in the demand for raw materials and the scarcity of resources is one of the reasons to move toward a circular economy. In this work, we propose the use of bagasse of *Agave tequilana* Weber var. Azul, which is a residue in the western region of Mexico, as a starting material for the preparation of added value products. This work contributes to the development of the circular economy, while avoiding the use of timber resources resulting from deforestation, providing solutions to two environmental problems in parallel, namely pollution and deforestation.

CNCs, bCNFs, and ubCNFs were obtained from *A. tequilana* Weber var. Azul bagasse by acid hydrolysis and mechanical shear, respectively. Thermoplastic starch biocomposites reinforced with 2.0 wt% and 6.0 wt% of CNCs and CNFs were prepared using the casting method. Recrystallization of amylose chains after the destructuring of native starch gave new crystallographic patterns Eh, Vh, and Va. The highest crystallinity values were obtained for the biocomposite films with 6.0 wt% reinforcement content. The chemical compatibility between glycerol and native starch in the biocomposite films was corroborated by SEM and FTIR, which was mainly attributed to hydrogen bonding between starch, glycerol, and nanocellulose. The water vapor permeability (WVP) decreased with the presence of CNCs. The solubility decreases with the presence of CNFs and CNCs. The roughness increased with the presence of nanofibers and decreased with the presence of nanocrystals. T_g_ and conductivity did not change drastically with composite content and type, but T_g_ diminished with moisture. The biocomposites tensile strength and modulus of elasticity increased with the addition of nanocellulose. The elasticity of the films decreased as the concentration of CNCs and CNFs increased. Biocomposite films prepared with ubCNFs showed the best tensile properties due to better adhesion of the nanofibrils with the matrix. CNFs and CNCs obtained from Agave bagasse are good reinforcement options for thermoplastic starch films. These findings contribute to overcoming the limitations of starch-based films by developing nanocomposite materials for future food packaging applications.

## Figures and Tables

**Table 1:** Crystallinity and color parameters of thermoplastic starch, CNC biocomposites, and bleached and unbleached CNF biocomposite films.

Biocomposite Film	L*	a*	b*	ΔE	Crystallinity (%)	Thickness (mm)	Electrical Conductivity (nA)
TPS	88.22 ± 2.91	0.15 ± 0.01	2.24 ± 0.06	---	8.8 ± 0.28	0.186 ± 0.007	10.36 ± 0.24
2.0 wt% ubCNF	64.61 ± 1.81	1.44 ± 0.03	0.57 ± 0.02	23.70 ± 1.10	6.9 ± 0.14	0.222 ± 0.012	11.64 ± 0.25
6.0 wt% ubCNF	54.23 ± 1.52	1.59 ± 0.05	0.86 ± 0.02	34.05 ± 1.39	7.1 ± 0.27	0.223 ± 0.008	12.20 ± 0.16
2.0 wt% bCNF	83.16 ± 2.01	−0.78 ± 0.03	1.65 ± 0.05	5.18 ± 0.92	5.6 ± 0.31	0.212 ± 0.013	10.79 ± 0.09
6.0 wt% bCNF	75.06 ± 2.48	2.63 ± 0.07	4.93 ± 0.12	13.66 ± 0.44	7.4 ± 0.28	0.207 ± 0.013	10.82 ± 0.07
2.0 wt% CNC	72.34 ± 1.74	2.55 ± 0.07	8.06 ± 0.27	17.08 ± 1.19	7.2 ± 0.31	0.175 ± 0.011	11.98 ± 0.11
6.0 wt% CNC	68.83 ± 1.93	3.98 ± 0.13	12.1 ± 0.29	22.07 ± 1.02	7.6 ± 0.29	0.181 ± 0.019	12.49 ± 0.20
pA	*p* < 0.05	*p* < 0.05	*p* < 0.05	*p* < 0.05	*p* < 0.05	*p* > 0.05	*p* < 0.05
pB	*p* < 0.05	*p* < 0.05	*p* < 0.05	*p* < 0.05	*p* < 0.05	*p* > 0.05	*p* < 0.05
pAB	*p* < 0.05	*p* < 0.05	*p* < 0.05	*p* < 0.05	*p* < 0.05	*p* > 0.05	*p* < 0.05

±Standard deviation; A Factor: composite concentration (2.0, 6.0 wt%); B Factor: composite type (ubCNF, bCNF, and CNC). pA, pB, pAB: *p* for A, B, and interaction between A and B, respectively. *p* < 0.05: A, B, or interactions between A and B are significantly different, *p* > 0.05: not significant.

**Table 2:** Ratios of peak areas from FTIR spectra of TPS and ubCNF-, bCNF-, and CNC-based composites. The analysis was performed using two specimens per sample.

Peak Area Ratios. AREA_λ_/AREA_λREF._ (λREF: 925, 1077 cm^−1^)								
λ/λ_REF_	Control (TPS)	Composites (2.0 wt%)			Composites (6.0 wt%)			*p* (Statistical)
		ubCNF	bCNF	CNC	ubCNF	bCNF	CNC	pA, pB, pA,B a = 0.05
1077/925	0.74 ± 0.01	0.67 ± 0.005	0.67 ± 0.01	0.66 ± 0.01	0.67 ± 0.02	0.66 ± 7 × 10^−5^	0.67 ± 0.02	*p* > a, *p* > a, *p* < a
861/925	0.94 ± 0.04	0.93 ± 0.004	0.89 ± 0.01	0.88 ± 0.004	0.89 ± 0.02	0.89 ± 0.01	0.86 ± 0.006	*p* < a, *p* < a, *p* < a
2929/925	0.825 ± 0.001	0.82 ± 0.01	0.84 ± 0.01	0.81 ± 0.01	0.81 ± 0.01	0.82 ± 0.01	0.86 ± 0.07	*p* > a, *p* > a, *p* > a
3280/925	4.2 ± 0.10	4.42 ± 0.01	3.89 ± 0.18	4.43 ± 0.13	4.26 ± 0.04	4.05 ± 0.01	4.15 ± 0.002	*p* > a, *p* < a, *p* < a
1573/925	0.026 ± 0.01	0.08 ± 0.006	0.03 ± 0.001	0.04 ± 0.004	0.09 ± 0.02	0.07 ± 7 × 10^−4^	0.05 ± 0.016	*p* < a, *p* < a, *p* > a
1020/925	0.90 ± 0.02	1.58 ± 0.04	1.02 ± 0.06	0.96 ± 0.15	1.58 ± 0.1	1.16 ± 0.13	0.96 ± 0.37	*p* > a, *p* < a, *p* > a
1341/925	0.28 ± 0.06	0.25 ± 0.01	0.16 ± 0.05	0.12 ± 0.009	0.13 ± 0.05	0.14 ± 0.009	0.12 ± 0.007	*p* < a, *p* < a, *p* > a
1415/925	0.26 ± 0.005	0.32 ± 0.01	0.29 ± 0.03	0.27 ± 0.004	0.29 ± 0.005	0.26 ± 0.001	0.25 ± 0.002	*p* < a, *p* < a, *p* > a
997/925	2.30 ± 0.15	1.53 ± 0.02	2.11 ± 0.01	2.31 ± 0.07	1.57 ± 0.07	1.89 ± 0.15	2.37 ± 0.05	*p* > a, *p* < a, *p* > a
1357/925	0.162 ± 0.008	0.09 ± 5 × 10^−4^	0.15 ± 0.07	0.180 ± 0.01	0.097 ± 0.04	0.136 ± 0.01	0.26 ± 0.01	*p* > a, *p* < a, *p* > a
1041/925	0.79 ± 0.03	0.70 ± 0.005	0.65 ± 0.04	0.76 ± 0.17	0.76 ± 0.01	0.68 ± 0.02	0.96 ± 0.20	*p* > a, *p* > a, *p* > a
1206/925	0.26 ± 0.003	0.29 ± 0.01	0.25 ± 0.01	0.30 ± 5 × 10^−4^	0.28 ± 0.02	0.26 ± 0.008	0.30 ± 0.003	*p* > a, *p* < a, *p* > a
1020/1077	1.21 ± 0.05	2.36 ± 0.07	1.53 ± 0.06	1.45 ± 0.21	2.38 ± 0.20	1.76 ± 0.20	1.44 ± 0.60	*p* > a, *p* < a, *p* > a

±Standard deviation, A Factor: composite concentration (2.0 and 6.0 wt%), B Factor: composite type (ubCNF, bCNF and, CNC). pA, pB, pAB: *p* for A, B, and interaction between A and B, respectively. *p* < 0.05: A, B or interactions between A and B are significantly different, *p* > 0.05: not significant.

**Table 3:** Thermal parameters, rugosity, solubility, and water vapor permeation results.

Biocomposite Film	Solubility (%)	Water Vapor Transmission (g/h m^2^)	Water Vapor Permeance 10^10^ (g/Pa m s)	T_g1_ (°C)	T_g2_ (°C)	T_s_–T_e_ (°C)	Ash Content (%)	Rugosity (µm)
Control (TPS)	50.57 ± 0.85	14.69 ± 0.95	2.992 ± 0.194	67.0 ± 4.0	118.7 ± 4.9	252–452	4.174	0.36 ± 0.1
2.0 wt% ubCNF	48.71 ± 0.82	11.46 ± 0.88	2.786 ± 0.214	58.9 ± 0.8	121.2 ± 2.7	-	-	1.56 ± 0.3
6.0 wt% ubCNF	47.72 ± 0.86	11.13 ± 0.62	2.841 ± 0.158	65.1 ± 2.3	115.3 ± 6.5	214–438	7.985	0.73 ± 0.08
2.0 wt% bCNF	47.86 ± 0.83	14.40 ± 0.28	3.501 ± 0.068	73.8 ± 5	121.6 ± 1.6	-	-	0.72 ± 0.33
6.0 wt% bCNF	46.98 ± 0.71	12.58 ± 0.42	2.990 ± 0.100	67.2 ± 4.3	117.9 ± 4.2	213–422	9.392	0.89 ± 0.27
2.0 wt% CNC	48.42± 0.98	12.69 ± 0.17	2.434 ± 0.032	70.6 ± 1.8	119.9 ± 2.6	-	-	0.26 ± 0.09
6.0 wt% CNC	47.57 ± 0.77	11.97 ± 0.08	2.373 ± 0.015	68.3 ± 2.1	118.9 ± 2.7	204–452	4.222	0.15 ± 0.002
pA	*p* < 0.05	*p* < 0.05	*p* < 0.05	*p* < 0.05	*p* < 0.05			*p* > 0.05
pB	*p* > 0.05	*p* < 0.05	*p* < 0.05	*p* < 0.05	*p* < 0.05			*p* > 0.05
pAB	*p* > 0.05	*p* > 0.05	*p* < 0.05	*p* < 0.05	*p* < 0.05			*p* < 0.05

T_s_–T_e_: start–end degradation temperature; ± standard deviation; A Factor: composite concentration (2.0, 6.0 wt%); B Factor: composite type (ubCNF, bCNF and, CNC). pA, pB, pAB: *p* for A, B, and interaction between A and B, respectively. *p* < 0.05: A, B, and interactions between A and B are significantly different, *p* > 0.05: not significant.

## Data Availability

The data that support the findings of this study are available from the corresponding authors, E.R.M.-B. and S.G.-E., upon reasonable request.

## Supplementary Materials

The following supporting information can be downloaded at: https://www.mdpi.com/article/10.3390/polym15183793/s1, Figure S1: 96-well plate used to determine the minimum inhibitory concentration.
