# Supplementary figures and images for: Thermoplastic Starch Biocomposite Films Reinforced with Nanocellulose from Agave tequilana Weber var. Azul Bagasse
Source: Polymers (Basel). 2023 Sep 17;15(18):3793. doi: 10.3390/polym15183793 (PMC10534575; doi:10.3390/polym15183793)

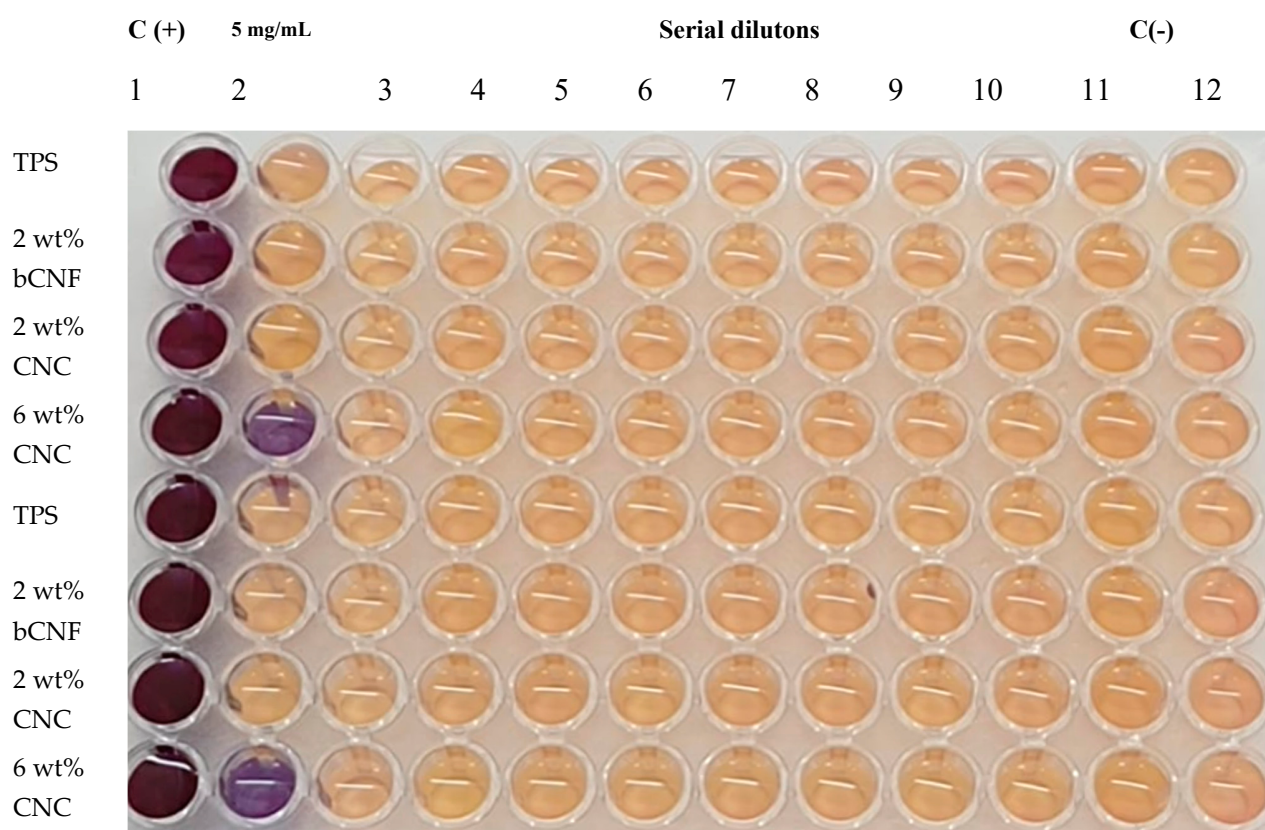

S1. 96-well plate used to determine the minimum inhibitory concentration

Supplement: Supplementary file 1 [file polymers-15-03793-s001.zip › polymers-2562562-supplementary.pdf]
